# Light as a Health Maker

**Published:** 1907-11

**Authors:** W. J. McGee

**Affiliations:** Former President American Anthropological Association


### Light as a Health Maker

By W. J. McGEE
Former President American Anthropological Association

(*New York Tribune Sunday Magazine*, September 22, 1907.)

LIGHT starvation is one of the most serious afflictions of the human race. It is indeed the fruitful cause of a multitude of physical ills. Strange does it seem that the function of light as a maker of health should have been ignored to so great an extent. Its hygienic usefulness seems to be almost unrecognized today by the medical faculty. A physician sends his patients to the sea shore, and at the end of a few weeks they come back to town with a gratifying augmentation of health and vigor. The improvement is attributed to the tonic effect of the salt air, and to the incidental change of diet and scene. In a measure this idea is correct; but the truth is that exposure to light has been the chief means of cure.

Light is nature's most important curative agency. It is often said that there is nothing like plenty of fresh air and exercise to make people well and keep them so; but, while this is true enough, the benefit obtained is due mainly to the incidental exposure to unlimited light. A game of tennis is a light bath; a row on the river is a light bath; a bath in the sea is at the same time a bath of light, which is one reason why it is so healthful.

Young people at the seaside try their best, as they say, to "get a coat of tan," for which purpose they expose themselves as much as possible to the sunshine; and when the tan has been acquired they exhibit it proudly as a sign of health. They do this without knowing the reason why, because tan, which is the result of free exposure to a glare of light, is associated in everybody's mind with physical vigor and "feeling good."

TAN INDICATES VIGOR.

It is true then that the augmentation of bodily vigor comes with the tan, because the latter is obtained only by exposure to baths of unlimited light. People who live in cities become pale and steadily paler of complexion, for lack of light. Incidentally through the operation of the same cause they lose health; the circulation of their blood becomes less vigorous; and the functioning of their vital organs less energetic. Though ignorant of the cause, they recognise the fact, and when summer comes again they say, "We must get away to the sea shore."

Or perhaps it may be to the mountains. At all events, it must be into the open somewhere. The seaside is usually preferred because of a notion that salt air is specially beneficial. So indeed it may be; but the main advantage of the sea shore is that there is unlimited light, with a maximum of temptation to be out in the sunshine all day long. Lying on the beach for hours together, one literally takes in health in every pore. The fewer clothes one is able to wear the better.

Dame Nature, when she brought the human race into existence, does not seem to have contemplated the possibility of civilised conditions. The first human beings dwelt in trees, and wore no clothing whatever. Under such circumstances they enjoyed the benefit of unlimited light. Civilised man, on the other hand, hides from the light in artificial caves constructed of baked mud or of stone, and in addition covers nearly all of his body with clothing that is practically light proof.

The theory here set forth is reducible to a basis of well understood fact. In plants (whose vitality, while not the same, is at least analogous to that of animals) the green coloring matter called chlorophyl is a chemical substance which enables the vegetable organism to utilise the energy of light. If there is no light, however, there will be no chlorophyl. A potato plant grown in a dark cellar will be not green, but white, with a long stem and no foliage that amounts to anything.

In human beings or other animals the red corpuscles of the blood appear to perform a function analogous to that of chlorophyl in plants,—enabling the body to utilise the energy of light in vital action. But if there is lack of light, these corpuscles become fewer, and the person thus afflicted is informed by the physician that he is suffering from anemia, a complaint of which the principal manifestations are pallor and a general lowering of the tone of the vitality. In the crowded slums of cities, where people are shut up most of the time in poorly lighted rooms, they are usually anemic, and the mortality among children is frightful.

### GIVE THEM MORE LIGHT.

All the efforts made to improve the condition of slum dwellers in the congested centers of population are in the direction of giving them more light, though the ostensible objects in view are to provide more fresh air and cleaner quarters. Undoubtedly fresh air and cleanliness are of great importance; but most essential of all is light. When through the aid of "fresh air funds" and other charitable means the children of the slums enjoy the advantage of a vacation in the country, it is the opportunity of unlimited light that does them more good than anything else.

While a coat of tan is regarded as accentuating feminine beauty, because it is the very hue of health, the girl who freckles is usually reluctant to expose herself to sunshine. It is a pity, because the freckles, like tan and for the same reason, are properly to be regarded as a sign of physical vigor. A young woman who is subject to freckles is likely to find that she feels better in proportion to the number of freckles she has. In the winter time of course she has comparatively few, because of relative nonexposure. If she wishes to get rid of them altogether, she can do so by remaining in the dark. But what would be the effect of such a course of procedure upon her health?

Mark Twain has remarked that we are not in truth white people, as we choose to call ourselves, but colored folks; and, he adds, the Negroes are "discolored." There is verity in the observation, inasmuch as our complexion is in reality of a pinkish yellow hue. This color is due to a pigment that is present in the layer of skin tissue immediately beneath the epidermis, or outer skin. Exposure to sunshine in some individuals causes the pigment to collect in little patches, visible through the transparent epidermis, which are called freckles.

### CONQUERING RACES ARE BLOND.

It cannot be said that freckles are beautifying. Yet there may be some consolation in the knowledge that they are almost peculiarly a characteristic of the blond type of human beings, which by some eminent anthropologists is declared to be the higher type, in evidence of which attention is called to the fact that the conquering races of the world have possessed light hair and blue eyes. But, whether this is correct or not, it is safe to fall back upon the statement that freckles and good health are likely to go together.

A recognition of the health giving powers of light is evinced by the recent introduction into medical practice of the electric light bath, in which the patient is exposed to a flood of electricity so powerful that if its rays were concentrated upon any one spot they would burn severely. This method of treatment has been adopted very successfully, it is said, in cases of internal trouble of various kinds, and particularly in the cure of digestive disorders.

Exposure to sunshine seems to have a remarkable efficiency as a tonic for the capillary bloodvessels. Long ago the efficacy of the sun bath, so called, for invalids was recognised, and at the present time resort is had to treatment of this kind on a considerable scale at some sanatoriums. Even the seaside hotels have taken up the idea, and the "sun pavilion" of glass is advertised as an attractive adjunct of many a salt water caravansary. Nor should it be forgotten that light in various forms, such as the Finsen light, is now being used with excellent results for the cure of skin and other diseases. In this connection it ought to be remembered that radiant energy akin to light has various effects, some of which are harmful. The *x*-rays, for example, will burn severely, and will even destroy hair. Electric light under certain circumstances will make people bald. Accordingly, precautions must be exercised.

The sun is the source not only of all light, but of all the energy of whatever description that is manifested on the earth. Our very bodies may be said to be built out of this sun derived energy, and the work done by our muscles and vital organs all proceeds from the same origin. When for instance we wind a clock, we store in a coiled spring some of the energy of a beefsteak that was built out of materials derived from grass grown by the rays of the sun.

Considering the matter from this point of view, it is reasonable to regard light as the source not only of our being, but of our well being. Who is there who does not feel better, more cheerful, and more energetic on a sunny day than when the sky is covered with clouds? On the other hand, what is so depressing to both body and mind as confinement in darkness or in a gloomy place? Indeed, it is all too obvious to need argument. Light, and plenty of it, is the first essential to our welfare. The more we have of it, the better we are. We need fresh air, good food, a reasonable amount of bodily exercise, and clean surroundings. But above all these things, as a necessity of existence, we must have light,—the prime source of cheerfulness and of health, both moral and physical.

